# Mitochondrial Metabolic Reprogramming and Immune Evasion in Colorectal Cancer: Research Advances and Mechanistic Perspectives

**DOI:** 10.3390/ijms27209131

**Published:** 2026-10-15

**Authors:** Da Ding, Jingyi Hu, Yitong Wang, Lei Jiang

**Affiliations:** The First School of Clinical Medicine, Lanzhou University, Lanzhou 730000, China

**Keywords:** colorectal cancer, mitochondrial metabolic reprogramming, metabolic plasticity, tumor microenvironment, mitochondrial quality control, immune evasion

## Abstract

Colorectal cancer (CRC) is one of the most prevalent malignancies of the gastrointestinal tract worldwide. Notably, most patients with microsatellite-stable (MSS) tumors show primary resistance to immune checkpoint inhibitors (ICIs), creating an unmet clinical need. The mechanisms underlying immune evasion and strategies to overcome therapeutic resistance have therefore become important topics in CRC immunology research. Recent studies indicate that mitochondrial metabolic reprogramming contributes to immune evasion in CRC. Tumor cells display metabolic plasticity through dynamic shifts between glycolysis and oxidative phosphorylation (OXPHOS), which can intensify the depletion of critical nutrients, particularly glucose and glutamine, within the tumor microenvironment (TME). These metabolic changes can also promote the accumulation of immunosuppressive metabolites, including lactate, thereby impairing the function of effector cells such as CD8^+^ T cells and dendritic cells (DCs) and supporting the expansion of regulatory T cells (Tregs) and myeloid-derived suppressor cells (MDSCs). Lactate may further reshape the immune landscape through hydroxycarboxylic acid receptor 1 (HCAR1) signaling and histone lactylation. In parallel, mitochondrial quality control can regulate tumor immunogenicity by influencing mtDNA availability and cGAS-STING signaling. Therapeutic approaches targeting glycolysis, glutamine metabolism, fatty acid oxidation, lactate transport, and mitochondrial dynamics have shown immune-sensitizing activity in preclinical models. Nanodelivery systems and gene-editing technologies may improve the spatial and cellular specificity of these interventions. This review summarizes how mitochondrial metabolic reprogramming contributes to immune evasion in CRC and evaluates mitochondria-directed strategies for improving treatment responsiveness, particularly in MSS CRC.

## 1. Introduction

Colorectal cancer (CRC) is one of the most prevalent and lethal malignancies worldwide and remains a major public health concern. According to the GLOBOCAN 2022 database, CRC ranks third in incidence and second in cancer-related mortality among all cancers [1]. Because early-stage symptoms are often nonspecific, approximately 20% of patients present with synchronous distant metastases at diagnosis; consequently, the five-year overall survival rate for patients with metastatic CRC remains below 20% [2].

In recent years, immune checkpoint inhibitors (ICIs) have achieved substantial clinical benefit in several solid tumors. In CRC, however, their efficacy is largely confined to patients with microsatellite instability-high (MSI-H) or mismatch repair-deficient (dMMR) tumors, which represent approximately 5–15% of CRC cases. In contrast, most microsatellite-stable (MSS) CRCs exhibit primary or acquired resistance to ICIs [3,4]. Understanding the mechanisms underlying immunotherapy resistance in MSS CRC and identifying rational sensitization strategies are therefore important clinical objectives [5,6]. Mitochondria are central regulators of cellular energy metabolism, and their functional state can influence tumor progression and the tumor immune microenvironment. Emerging evidence indicates that mitochondrial metabolic reprogramming in cancer cells not only supports bioenergetic and biosynthetic demands but can also reshape the TME through interconnected metabolic and immune mechanisms [7,8].

CRC cells display metabolic plasticity and can adapt between glycolysis and oxidative phosphorylation (OXPHOS). This capacity promotes tumor-cell survival under nutrient-limited conditions and can impair antitumor immune cells, including CD8^+^ T lymphocytes and dendritic cells (DCs), through competitive depletion of nutrients such as glucose and glutamine and through accumulation of metabolites such as lactate and reactive oxygen species (ROS) [9]. In addition, mitochondrial quality control, including mitochondrial dynamics, biogenesis, and mitophagy, can influence tumor immunogenicity by regulating mitochondrial integrity, antigen-presentation capacity, and the release of damage-associated molecular patterns (DAMPs) [10].

This review summarizes current evidence on how mitochondrial metabolic reprogramming contributes to immune evasion in CRC and evaluates therapeutic strategies targeting mitochondrial metabolism to improve treatment responsiveness. Particular attention is given to metabolic interactions between tumor and immune cells, mitochondrial quality control, and mitochondria-associated immune signaling.

To identify relevant evidence, a structured literature search was conducted in PubMed and Web of Science on 17 September 2026. The principal search terms combined (“colorectal cancer” OR “CRC” OR “colon cancer”) with terms related to mitochondrial biology (“mitochondrial metabolism,” “mitochondrial metabolic reprogramming,” “oxidative phosphorylation,” “glycolysis,” “mitophagy,” “mitochondrial dynamics,” “mtDNA,” or “mitochondria-associated membranes”) and immune regulation (“immune evasion,” “tumor microenvironment,” “T cell,” “dendritic cell,” “macrophage,” or “immunotherapy”). Studies published during the preceding 10 years were prioritized, while earlier seminal studies were retained where necessary. Titles and abstracts were screened for direct relevance to CRC metabolism, mitochondrial quality control, immune regulation, or treatment response. Priority was given to original studies that identified the relevant cellular compartment and experimental model and provided mechanistic or translational evidence. Reviews were used for background context and to identify additional primary studies; non-CRC studies were retained only when they provided foundational mechanistic evidence and were identified as such in the text.

The mechanisms reviewed here should also be interpreted in the context of CRC heterogeneity. The consensus molecular subtype framework distinguishes CMS1 tumors with microsatellite instability and immune activation, CMS2 tumors with canonical WNT and MYC signaling, CMS3 tumors with prominent metabolic dysregulation, and CMS4 tumors with mesenchymal, stromal, and angiogenic features [11]. These differences suggest that mitochondrial and immune phenotypes may not be uniform across CRC subtypes, although direct evidence linking individual mitochondrial programs to CMS-specific treatment responses remains limited. Primary tumor location provides an additional source of variation: the frequencies of molecular alterations and CMS classes change along the proximal-to-distal colorectum, and a binary right- versus left-sided classification may not capture the full biological continuum [12]. Primary tumors and liver metastases also differ in their stromal and immune contexts. Single-cell and spatial analyses of matched CRC and liver metastases have identified extensive remodeling of the metastatic immune microenvironment, including metabolically active immunosuppressive macrophage states and treatment-associated changes in immune composition [13]. Findings obtained from a particular molecular subtype, anatomical site, or experimental model should therefore be generalized cautiously across CRC settings.

Accordingly, the following sections examine how metabolic reprogramming and nutrient competition alter immune-cell fitness, and how mitochondrial quality control, MAMs, and mtDNA-dependent signaling regulate tumor immunogenicity. These interconnected processes are discussed across tumor, immune, and stromal compartments to clarify their respective contributions to immune evasion in CRC.

## 2. Mitochondrial Metabolic Reprogramming and Metabolic Plasticity

### 2.1. Dynamic Balance Between Glycolysis and Mitochondrial Oxidative Phosphorylation

CRC cells can redistribute pyruvate between mitochondrial oxidation and cytosolic lactate production according to nutrient availability, oxygen supply, and oncogenic signaling. Under conditions that support mitochondrial oxidation, pyruvate is transported into mitochondria by the mitochondrial pyruvate carrier, composed of MPC1 and MPC2, and converted to acetyl-CoA by the pyruvate dehydrogenase complex. Acetyl-CoA subsequently enters the tricarboxylic acid cycle and supports oxidative phosphorylation and biosynthesis. Reduced MPC1 or MPC2 expression has been reported in subsets of CRC and can restrict mitochondrial pyruvate utilization, increase the availability of pyruvate for lactate production, and favor greater reliance on glycolysis [14]. However, CRC cells generally retain mitochondrial respiratory capacity and may use glucose-derived pyruvate, glutamine, fatty acids, or other substrates to support the tricarboxylic acid cycle. Stable-isotope tracing further showed that glutamine-derived carbon entered the TCA cycle in subcutaneous, orthotopic, patient-derived xenograft, and genetically engineered CRC models. Relative glutamine anaplerosis was greater in tumors than in adjacent normal colon tissues and was particularly evident in some PIK3CA-mutant CRC models [15]. The resulting coexistence of glycolysis and oxidative metabolism enables CRC cells to adjust their metabolic state in response to regional differences in oxygen and nutrient availability.

Retention of oxidative metabolism has functional consequences for CRC progression and treatment response. Increased PGC-1α activity can promote mitochondrial biogenesis and oxidative phosphorylation and has been associated with resistance to oxaliplatin [16], whereas subsets of colorectal cancer stem-like cells use mitochondrial respiration to maintain survival and stemness [17]. At the same time, increased expression of glycolytic enzymes, including hexokinase 2 (HK2), pyruvate kinase M2 (PKM2), and lactate dehydrogenase A (LDHA), supports glycolytic flux in many CRC cells [18,19,20]. The relative contribution of these pathways varies among tumor-cell states and can change during treatment or nutrient stress.

Metabolic plasticity also involves exchange of metabolites between malignant and stromal cells. Glycolytic cancer-associated fibroblasts can release lactate through monocarboxylate transporters. Where the appropriate transporter and enzymatic machinery are present, neighboring CRC cells may import extracellular lactate, convert it to pyruvate through LDHB, and use the resulting carbon in the tricarboxylic acid cycle and oxidative phosphorylation [21,22]. Direct metabolic evidence for this capacity was obtained using ^13C-lactate tracing in CRC cells. Inhibition of phosphoenolpyruvate carboxykinase 1 (PCK1) reduced the incorporation of lactate-derived carbon into the TCA cycle and decreased mitochondrial respiration, ATP production, and tumor growth in cell-based and mouse models [23]. This form of epithelial–stromal metabolic coupling can support mitochondrial metabolism in selected tumor regions, although the direction and magnitude of lactate exchange depend on transporter expression, substrate gradients, oxygen availability, and the metabolic states of the interacting cells.

Fatty acid oxidation provides an additional source of acetyl-CoA and reducing equivalents for mitochondrial metabolism. In CRC cells with sufficient fatty-acid uptake and oxidative capacity, this pathway can supplement glucose- and glutamine-derived carbon, support mitochondrial membrane potential, and contribute to redox homeostasis during changes in nutrient availability [24,25,26]. The ability to use several carbon sources allows CRC cells to maintain energy production when the availability of a single substrate becomes limiting. This metabolic flexibility also changes the extracellular environment encountered by immune cells: increased tumor-cell consumption of glucose and amino acids can restrict nutrient availability, whereas the release of lactate and other metabolites can alter immune-cell signaling and mitochondrial fitness [27,28]. These immune consequences are examined in greater detail in the following sections.

Together, these findings indicate that mitochondrial metabolic reprogramming in CRC is characterized by variable use of glycolysis, oxidative phosphorylation, and alternative carbon sources rather than by a uniform loss of mitochondrial respiration. This metabolic flexibility supports tumor-cell adaptation to spatial and treatment-associated changes in nutrient availability. It also alters the tumor microenvironment through nutrient consumption, metabolite release, and metabolic exchange with stromal cells, thereby creating conditions that can influence the survival and function of infiltrating immune cells. Among these extracellular consequences, lactate accumulation and acidification represent an important connection between tumor-cell carbon metabolism and immune regulation.

### 2.2. Lactate Metabolism and Extracellular Acidification in CRC

Mitochondrial dysfunction and lactate accumulation frequently coexist in colorectal cancer (CRC), but their relationship is context-dependent. Lactate production is primarily determined by glycolytic flux, pyruvate-to-lactate conversion, and cellular redox balance. Impaired oxidative phosphorylation may increase glycolytic dependence under specific conditions, thereby contributing to lactate accumulation, rather than acting as a direct and universal cause of this process [29]. Metabolomic analyses of paired human CRC and adjacent non-tumor tissues have identified increased lactate abundance in tumor tissues [30,31]. In CRC, lactate accumulation commonly occurs together with extracellular acidification and contributes to the formation of an acidic tumor microenvironment. However, the extent of acidification is also influenced by lactate transport, proton handling, local perfusion, and buffering capacity [32,33].

Extracellular lactate can be transported through monocarboxylate transporters, including MCT1 and MCT4, often in association with CD147. In CRC, preclinical evidence indicates that this lactate-transport axis can influence antitumor immunity. In human CRC spheroid–leukocyte co-cultures, MCT4 blockade enhanced immune-cell infiltration and T-cell function; in MC38 mouse models, its combination with PD-L1 blockade increased intratumoral pH, improved T-cell activation, delayed tumor growth, and prolonged survival [34]. In colorectal liver metastases, elevated lactate and extracellular acidification were associated with intracellular acidification, mitochondrial stress, and apoptosis of liver-resident NK cells in patient samples and ex vivo experiments [35]. These studies illustrate two related consequences of extracellular lactate accumulation: MCT-dependent lactate transport can influence immune-cell access and activation within primary CRC, whereas lactate-associated acidification and mitochondrial stress can directly impair NK-cell viability in the metastatic liver microenvironment. The relevant mechanism therefore depends on both the immune-cell population and the anatomical site examined.

Lactate may also act as a signaling metabolite that reshapes the immune microenvironment through receptor-dependent pathways. In CRC, HCAR1, also known as GPR81, can be activated by extracellular lactate. He et al. [36] reported that HCAR1 signaling increased CCL2 and CCL7 expression in CRC cells and promoted the recruitment of immunosuppressive CCR2^+^ polymorphonuclear myeloid-derived suppressor cells (PMN-MDSCs). In mouse models of CRC, Hcar1 deletion or reserpine treatment reduced PMN-MDSC recruitment, enhanced CD8^+^ T-cell-dependent antitumor immunity, and improved the response to PD-1 blockade. These findings support HCAR1 as a potential mediator of lactate-associated immune suppression; however, the therapeutic activity of reserpine in CRC remains preclinical. Single-cell and spatial transcriptomic analyses have also identified glycolysis-enriched tumor-cell subsets in MSI-H CRC that are associated with lactate-related immune remodeling, including Treg-related and macrophage-related transcriptional programs [37].

Lactate may further influence immune regulation through lysine lactylation. In MSI-H CRC, Zhang et al. [38] reported that MNDA-associated H3K18 lactylation at the CXCR2 promoter promoted PMN-MDSC recruitment and reduced sensitivity to PD-1 blockade in experimental models. This study provides a CRC-specific example of lactylation-associated immune regulation. Overall, lactate-associated receptor signaling and lactylation offer complementary mechanisms through which metabolic remodeling can reinforce immunosuppressive programs in CRC.

Lan et al. integrated single-cell transcriptomic, spatial transcriptomic, and bulk sequencing datasets to characterize lactate-related metabolic heterogeneity in CRC [39]. They developed a four-gene lactate metabolism score (LMscore), and a high LMscore was associated with shorter overall survival and a cold immune phenotype characterized by lower effector T-cell infiltration and enrichment of M2 macrophage- and cancer-associated fibroblast-related features. Spatial analyses further showed colocalization between lactate-active epithelial cells and fibroblasts. These findings support an association between lactate-related metabolic programs and an immunosuppressive CRC microenvironment.

**Figure 1:**
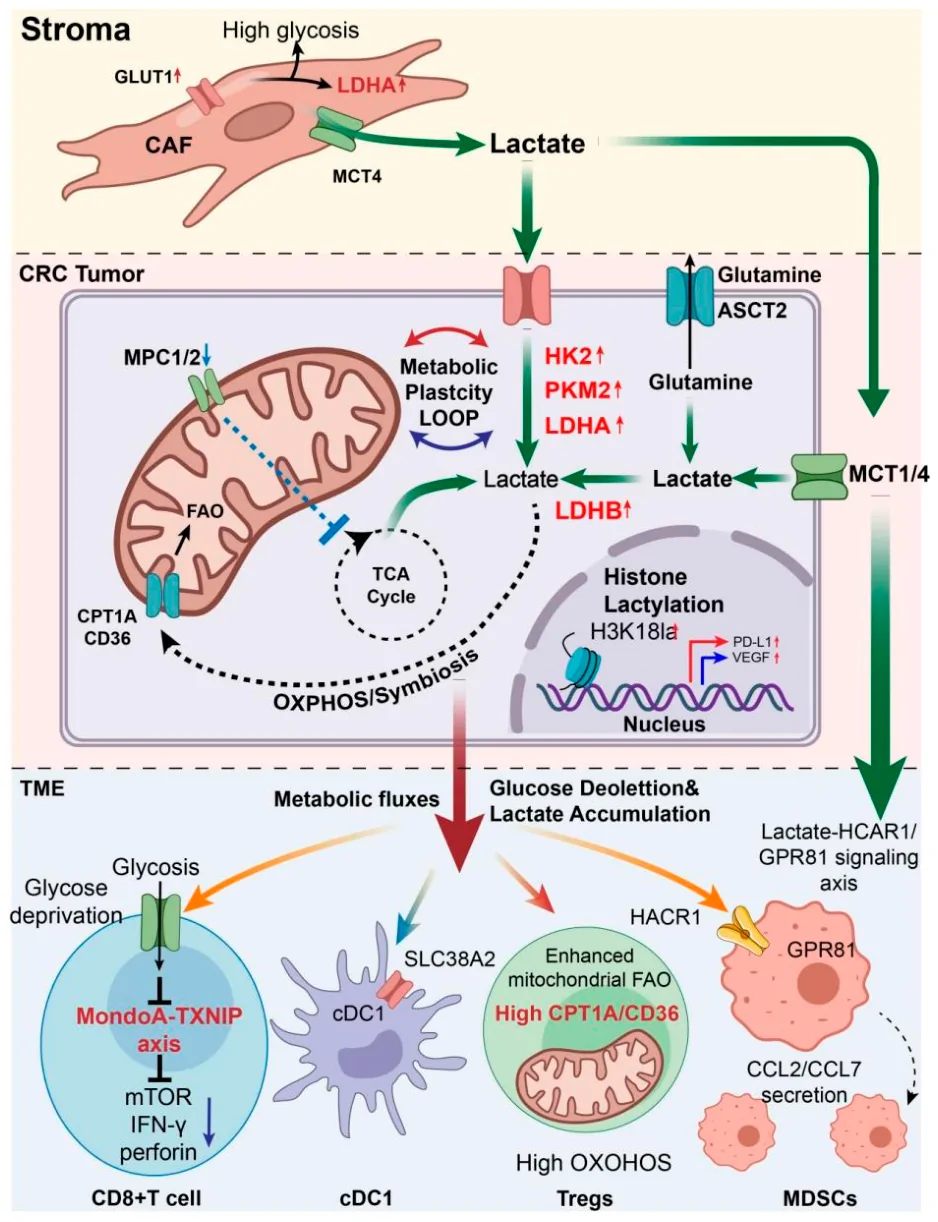
Mitochondrial metabolic reprogramming and immune evasion in CRC. Metabolic plasticity, nutrient competition, lactate signaling, and fatty acid oxidation impair CD8^+^ T-cell and cDC1 function while supporting Tregs and MDSCs. Arrows indicate flow or signaling; T-bars, inhibition; ↑/↓, increases/decreases. Colors distinguish compartments and components.

Taken together, lactate-associated immune regulation in CRC involves several interconnected processes, including altered pyruvate handling, lactate production and transport, extracellular acidification, HCAR1-dependent signaling, and lactylation-associated transcriptional regulation. These processes can affect tumor cells, stromal cells, and multiple immune-cell populations through distinct mechanisms. Accordingly, therapeutic interventions may target lactate production, extracellular transport, receptor-mediated sensing, or downstream epigenetic regulation; representative approaches and their supporting experimental models are compared in Section 5. The broader relationships among glycolysis, oxidative phosphorylation, lactate metabolism, and immune regulation are summarized in Figure 1.

## 3. Mitochondrial Metabolic Reprogramming and Metabolic Competition with Immune Cells

### 3.1. Glucose Competition and T-Cell Functional Suppression

CRC cells frequently exhibit increased glucose uptake and glycolytic activity. Mitochondrial perturbations, including impaired pyruvate import through the mitochondrial pyruvate carrier (MPC) or altered tricarboxylic acid-cycle activity, may increase reliance on glycolysis in specific metabolic contexts. This shift can be accompanied by increased GLUT1 expression and enhanced glucose consumption [14,18,19,20]. Within a nutrient-limited tumor microenvironment, high tumor-cell glucose utilization can reduce glucose availability for infiltrating immune cells. Thus, mitochondrial metabolic reprogramming may contribute to glucose competition by altering carbon utilization in CRC cells and reshaping the metabolic conditions under which T cells maintain their effector function.

Upon activation, CD8^+^ T cells undergo metabolic reprogramming that increases glycolytic flux while maintaining mitochondrial metabolism to support ATP production, biosynthesis, and effector differentiation. In glucose-limited environments, reduced nutrient availability can attenuate mTORC1 signaling and decrease interferon-γ and granzyme B production [27,40,41]. Prolonged glucose restriction may therefore impair T-cell proliferation, cytokine secretion, and target-cell killing, thereby contributing to T-cell dysfunction within the tumor microenvironment.

At the molecular level, mitochondrial ROS may stabilize HIF-1α and promote the expression of GLUT1 and glycolytic enzymes, thereby increasing glucose uptake and glycolytic flux in CRC cells [42]. This response provides one mechanism through which mitochondrial stress can reinforce tumor-cell glucose consumption and intensify metabolic competition within the tumor microenvironment. In addition to direct glucose competition, lactate-related signaling may further impair glucose utilization by effector T cells. In experimental systems, lactate uptake by CD8^+^ T cells has been linked to activation of the SENP1–MondoA–TXNIP axis, which restricts GLUT1-dependent glucose uptake and weakens T-cell effector function [28]. This mechanism provides a potential connection between a lactate-rich microenvironment and reduced metabolic fitness of antitumor T cells.

Metabolic competition in CRC also involves stromal cells. Evidence from CRC organoid–CAF co-culture models indicates that cancer-associated fibroblasts can reinforce the nutrient-limited and immunosuppressive conditions generated by tumor-cell metabolic reprogramming. Strating et al. found that glycolytic CAFs displayed hypoxia- and immunosuppression-related transcriptional programs, whereas co-culture-conditioned media contained increased lactate, TGF-β1, and VEGFA and inhibited T-cell proliferation [43]. More recently, Peng et al. reported that elevated glycolysis, intracellular lactate, and H3K18 lactylation in CAFs promoted extracellular-matrix remodeling and limited CD8^+^ T-cell infiltration in CRC models [44]. Together, tumor-cell and stromal-cell metabolic programs can establish a nutrient-limited microenvironment that constrains effector T-cell function and favors immune suppression.

Collectively, glucose competition in CRC reflects coordinated metabolic interactions among tumor cells, stromal cells, and infiltrating immune cells. Increased glucose consumption and lactate-associated metabolic remodeling can limit the metabolic fitness of effector T cells while supporting an immune-restrictive microenvironment. This framework provides a basis for considering additional nutrient-competition mechanisms, particularly glutamine metabolism, which can further influence the function of antigen-presenting cells and antitumor lymphocytes.

### 3.2. Glutamine Competition and Dendritic Cell Dysfunction

Glutamine metabolism is an important component of mitochondrial metabolic reprogramming in CRC. Following uptake, glutamine is converted to glutamate and subsequently to α-ketoglutarate, which enters the mitochondrial tricarboxylic acid cycle and provides anaplerotic support for oxidative metabolism, redox homeostasis, and biosynthetic processes. Increased expression of glutamine transporters, including ASCT2/SLC1A5, has been reported in subsets of CRC, indicating that tumor cells may acquire glutamine efficiently within the tumor microenvironment [45,46]. Through this mitochondrial glutamine-utilization program, CRC cells can sustain metabolic flexibility while reducing glutamine availability for surrounding immune cells.

A major immune consequence of tumor-cell glutamine consumption is impaired cDC1 function. cDC1s capture tumor-associated antigens, cross-present them through MHC class I molecules, and prime cytotoxic CD8^+^ T-cell responses. In experimental models, glutamine restriction reduces cDC1 maturation and decreases the expression of CD86, MHC class II, and IL-12, thereby weakening antigen presentation and T-cell priming [47]. Thus, competition for glutamine links mitochondrial nutrient utilization by CRC cells to defective initiation of adaptive antitumor immunity.

This interaction is mediated in part by SLC38A2-dependent glutamine acquisition. In MC38 CRC models, tumor cells expressed higher levels of SLC38A2 than intratumoral cDC1s. Under low-glutamine conditions, exposure of cDC1s to MC38-conditioned medium reduced their maturation and capacity to stimulate T-cell proliferation, whereas glutamine supplementation restored these functional defects [47]. Within cDC1s, glutamine availability promotes formation of the FLCN–FNIP2 complex and restrains TFEB activity, supporting the transcriptional programs required for antigen presentation and effector CD8^+^ T-cell responses. Accordingly, DC-specific deletion of Slc38a2 or Flcn impaired cDC1-dependent antitumor immunity and eliminated the benefit of intratumoral glutamine supplementation in mouse tumor models [47]. The SLC38A2–FLCN–TFEB axis therefore provides a defined mechanism through which tumor-cell glutamine utilization can compromise dendritic-cell function.

Glutamine competition should be considered alongside other amino-acid pathways that contribute to immune suppression in CRC. For example, tryptophan catabolism through IDO1 or TDO generates kynurenine and can further limit antitumor immune responses [48,49]. Together, these findings connect mitochondrial glutamine utilization in CRC cells with defective cDC1-mediated antigen presentation. Preferential glutamine acquisition by tumor cells restricts the extracellular glutamine available to cDC1s, while reduced SLC38A2–FLCN–TFEB signaling within cDC1s weakens the transcriptional and metabolic programs required for their maturation and cross-presentation. The resulting impairment of CD8^+^ T-cell priming provides a mechanistic link between tumor-cell amino-acid metabolism and reduced adaptive antitumor immunity [47].

### 3.3. Fatty Acid Metabolism and the Advantage of Regulatory T Cells

Lipid availability and mitochondrial fatty acid metabolism can provide tumor-infiltrating regulatory T cells (Tregs) with a survival advantage in CRC. Whereas activated effector T cells require substantial glycolytic flux to sustain rapid proliferation and cytotoxic activity, Tregs can adapt to nutrient-limited tumor conditions through lipid uptake and mitochondrial oxidative metabolism. In MC38 colon carcinoma models, intratumoral Tregs upregulated the fatty-acid scavenger receptor CD36. CD36-mediated lipid uptake activated PPAR-β signaling, supported mitochondrial fitness and NAD^+^/NADH balance, and promoted Treg persistence and suppressive activity within the tumor microenvironment [50]. This metabolic adaptation may allow Tregs to remain functional under conditions in which glucose availability and effector T-cell activity are constrained.

Tumor-cell-derived signals may further reinforce the accumulation of suppressive Treg states in CRC. Zi et al. reported that increased SIRT1 expression in CRC cells enhanced CX3CL1 secretion, and CX3CL1–CX3CR1 signaling promoted the transition of TCF7^+^ Tregs toward TNFRSF9^+^ Tregs through SATB1 and BTG2 activation [51]. These TNFRSF9^+^ Tregs displayed enhanced suppressive features and were associated with CD8^+^ T-cell exhaustion. This signaling axis may cooperate with, but is mechanistically distinct from, the CD36-dependent lipid-uptake program that supports Treg mitochondrial fitness.

Fatty acid oxidation also supports an immunosuppressive phenotype in tumor-associated plasmacytoid dendritic cells (TA-pDCs). In colon-cancer specimens and mouse models, PPARγ signaling increased CPT1A expression in TA-pDCs and enhanced fatty acid oxidation [52]. CPT1A-dependent metabolic reprogramming maintained an immunosuppressive pDC phenotype characterized by increased ICOSL expression and reduced OX40L, interferon-α, and tumor necrosis factor-α production. CPT1A inhibition reduced pDC-mediated Treg generation, improved CD8^+^ T-cell function, and enhanced the antitumor activity of combined ICOSL and PD-L1 blockade in murine colon cancer models [52].

## 4. Mitochondrial Quality Control and Immunogenicity Regulation

### 4.1. Mitophagy and mtDNA-Dependent Innate Immune Signaling

Mitophagy is a mitochondrial quality-control process that selectively removes damaged mitochondria and limits the accumulation of mitochondrial reactive oxygen species (mtROS) and mitochondrial DNA (mtDNA) [53]. This process is closely linked to tumor immunogenicity because damaged mitochondria can release mtDNA into the cytosol. Cytosolic mtDNA activates cGAS, promotes cGAMP production, and initiates STING–TBK1–IRF3 signaling, which can induce type I interferon responses [10]. In tumor cells, mitophagy may restrict this mtDNA-dependent innate immune signal by eliminating damaged mitochondria. In immune cells, however, mitophagy can preserve mitochondrial fitness and support the functional competence of dendritic cells and T cells [54]. Thus, mitophagy acts as a context-dependent regulator of mitochondrial danger signaling.

The PINK1–Parkin pathway is a canonical ubiquitin-dependent mechanism of mitophagy. Following mitochondrial depolarization, PINK1 accumulates on the outer mitochondrial membrane and promotes Parkin-dependent ubiquitination of mitochondrial proteins, allowing the autophagic machinery to recognize and remove damaged organelles [53]. Mitophagy can also proceed through receptor-dependent pathways, including BNIP3, NIX/BNIP3L, and FUNDC1, which directly bind LC3 through LC3-interacting regions. These pathways respond to distinct forms of mitochondrial stress, such as hypoxia, oxidative stress, and impaired protein import. In CRC, the mitochondria-localized peptide LRRC75A-AS1-ORF3 has been reported to promote mitophagy, reduce cytosolic mtDNA abundance, and attenuate cGAS–STING signaling, thereby facilitating immune escape in experimental models [55]. This finding illustrates how enhanced tumor-cell mitophagy can limit mtDNA-dependent innate immune sensing.

Conversely, unresolved mitochondrial damage can increase the availability of mtDNA for cytosolic DNA sensing. In CRC cells, serine deprivation disrupts mitochondrial network integrity and promotes the accumulation of mtDNA in the cytosol. Cytosolic mtDNA is recognized by cGAS, which generates cGAMP and activates the STING–TBK1–IRF3 pathway, leading to type I interferon production. Depletion of mtDNA or blockade of mtDNA release attenuated cGAS–STING activation during serine restriction, demonstrating that mtDNA leakage is required for this response. In immunocompetent CRC models, serine restriction enhanced intratumoral immune-cell infiltration and suppressed tumor growth; these effects were weakened by T-cell depletion or IFN receptor blockade [56].

### 4.2. Mitochondrial Dynamics and MAMs

Mitochondrial dynamics and mitochondria-associated endoplasmic reticulum membranes (MAMs) regulate mitochondrial function at two interconnected levels. Mitochondrial fission and fusion determine organelle morphology, distribution, respiratory capacity, and the segregation of damaged mitochondrial components. Fission is primarily mediated by dynamin-related protein 1 (DRP1), whereas fusion depends on mitofusin 1/2 (MFN1/2) at the outer mitochondrial membrane and optic atrophy 1 (OPA1) at the inner mitochondrial membrane [57]. MAMs are specialized ER–mitochondria contact sites that coordinate Ca^2+^ transfer, phospholipid exchange, endoplasmic-reticulum stress responses, and mitochondrial metabolism [58].

In KRAS-mutant CRC, oncogenic KRAS activates the MEK/ERK pathway and promotes phosphorylation of DRP1 at serine 616. This persistent DRP1 activation increases mitochondrial fragmentation and reduces the mitochondrial DNA pool available for radiotherapy-induced cytosolic release. As a result, radiotherapy-induced cGAS–STING activation and type I interferon production are attenuated, limiting the immunogenic consequences of DNA damage. In preclinical CRC models, DRP1 inhibition restored mtDNA-dependent innate immune signaling and enhanced radiotherapy-associated antitumor immunity [59].

Fusion-related mechanisms differ according to cellular compartment. In CRC cells, reduced METTL14 expression increases pri-miR-17 stability through an m6A–YTHDC2-dependent mechanism, thereby increasing miR-17-5p, which directly targets the 3′ untranslated region of MFN2 [60]. In tumor-infiltrating CD8^+^ T cells, MFN2 interacts with SERCA2 at ER–mitochondria contact sites, supports controlled mitochondrial Ca^2+^ uptake, and prevents excessive Ca^2+^-induced mitochondrial dysfunction. MFN2 deficiency reduces mitochondrial respiration, ATP generation, and cytokine production, thereby impairing CD8^+^ T-cell effector function in tumor models [61].

OPA1 provides a fusion-associated mechanism linking mitochondrial fitness to antigen presentation in conventional type 1 dendritic cells (cDC1s) [57,62]. OPA1 maintains inner mitochondrial membrane organization and supports respiratory-chain integrity through an OPA1–NRF1 regulatory axis. Loss of OPA1 reduces NRF1 expression, impairs oxidative phosphorylation, and lowers the cellular NAD^+^/NADH ratio. These metabolic changes activate AMPK-associated autophagic–lysosomal pathways and accelerate degradation of the MHC class I–peptide pool [62,63], thereby impairing the cross-presentation of tumor antigens. Consistent with this mechanism, OPA1-deficient cDC1s showed a reduced capacity to stimulate antigen-specific OT-I-cell proliferation. In vivo, dendritic-cell-specific Opa1 deficiency accelerated the growth of MC38 colon adenocarcinoma and was accompanied by reduced intratumoral CD8^+^ T-cell accumulation, interferon-γ production, and granzyme B expression [62]. These findings connect mitochondrial inner-membrane organization and respiratory fitness in cDC1s with the maintenance of antigen cross-presentation and CD8^+^ T-cell-mediated tumor control. Beyond mitochondrial architecture itself, mitochondrial function is further shaped by physical contacts with the endoplasmic reticulum, particularly through mitochondria-associated membranes (MAMs).

MAMs coordinate mitochondrial metabolism partly by controlling Ca^2+^ transfer from the endoplasmic reticulum. IP3R releases Ca^2+^ from the ER lumen, GRP75 couples IP3R to VDAC1 at the outer mitochondrial membrane, and MCU mediates Ca^2+^ entry into the mitochondrial matrix [58,64]. This localized Ca^2+^ transfer regulates the activity of tricarboxylic acid-cycle enzymes, oxidative phosphorylation, reactive oxygen species production, and mitochondrial permeability. Alterations in the abundance or organization of these contact-site proteins can therefore change mitochondrial bioenergetics and stress signaling in CRC cells [64]. However, the direct contribution of individual MAM Ca^2+^-transfer complexes to immune evasion in CRC remains less well defined than their established roles in tumor-cell survival and metabolic adaptation.

MAMs also provide sites for phospholipid transfer and remodeling, creating a connection between membrane lipid composition and mitochondria-associated immune signaling [58,64]. In CRC, ACSL4-dependent activation of long-chain polyunsaturated fatty acids has been linked to suppression of the mitochondrial RIG-I–MAVS pathway. Genetic or pharmacological reduction of ACSL4 increased type I interferon signaling, CXCL10 and CXCL11 expression, and antigen-presentation-related genes in CRC cells. In immunocompetent mouse models, ACSL4-deficient tumors showed increased CD3^+^ and CD8^+^ T-cell infiltration and greater sensitivity to PD-L1 blockade [65]. These findings connect lipid remodeling with mitochondrial antiviral-signaling machinery in CRC, although whether this immune phenotype is directly mediated by altered MAM structure requires further investigation.

### 4.3. Immune Activation by Mitochondrial DNA: A Dual Regulatory Role

The immunological effect of mtDNA in CRC depends on whether mitochondrial stress permits mtDNA to reach the cytosol of CRC cells [10,53]. Cytosolic mtDNA is recognized by cGAS, which produces cGAMP and activates the STING–TBK1–IRF3 pathway, leading to type I interferon production and expression of interferon-stimulated genes [66]. Activation of cGAS–STING within CRC cells can promote antitumor immunity indirectly by inducing type I interferons and chemokines that recruit and activate antigen-presenting cells, natural killer cells, and effector T cells within the tumor microenvironment [53].

Several distinct forms of mtDNA alteration should be considered when interpreting mitochondrial immune signaling in CRC. Inherited mtDNA polymorphisms are population-level variants that may define mitochondrial haplotypes but do not necessarily impair mitochondrial function [67,68]. By contrast, somatic mtDNA mutations arise within tumor cells and may affect respiratory-chain genes, mitochondrial transfer RNAs, or ribosomal RNAs. Their functional consequences depend on the affected locus and the proportion of mutant mtDNA molecules, or heteroplasmy, within the cell. Changes in mtDNA copy number, methylation, oxidative damage, and cytosolic release represent additional layers of mitochondrial dysregulation and should not be treated as equivalent to sequence mutations [67,69]. In the immune context discussed here, the amount, integrity, oxidation state, and subcellular localization of mtDNA may be as important as its nucleotide sequence, because these properties influence its accessibility to cytosolic DNA-sensing pathways [69].

In experimental CRC models, ACADS was reported to regulate mito-DNMT1-dependent mtDNA methylation and the availability of mtDNA in the cytosol. ACADS interacts with mito-DNMT1 and promotes its degradation, thereby reducing mito-DNMT1-dependent mtDNA methylation and facilitating mtDNA leakage. Conversely, ACADS deficiency increases mtDNA methylation and the expression of mtDNA repair-associated proteins, reduces cytosolic mtDNA abundance, and attenuates cGAS–STING signaling. In CRC mouse models, ACADS loss was associated with reduced interferon-related signaling, an immunosuppressive tumor microenvironment, and accelerated tumor progression [70].

Mitochondrial membrane injury provides an additional route for mtDNA release. In CT26 mouse CRC cells, experimental overexpression of DNA-dependent activator of interferon-regulatory factors (DAI), also known as Z-DNA-binding protein 1 (ZBP1), promoted ferroptosis, mitochondrial oxidative stress, and cytosolic accumulation of mtDNA. These changes were accompanied by activation of the cGAS–STING pathway. In conditioned-medium experiments, DAI-overexpressing CT26 cells promoted M1-like features and reduced M2-like polarization in RAW264.7 macrophages; corresponding changes in macrophage polarization and tumor growth were also reported in mouse models [71]. This evidence links DAI-induced ferroptotic stress in CRC cells with mtDNA-dependent innate immune signaling and macrophage reprogramming, although it derives from experimentally manipulated mouse cell systems.

A different route of mitochondrial membrane injury was reported in CT26 cells treated with andrographolide. Andrographolide promoted VDAC-associated mitochondrial membrane depolarization and mitochondrial permeability transition-pore opening, resulting in the cytosolic release of oxidized mtDNA. Activation of the cGAS–STING–IFN-β pathway was accompanied by increased dendritic-cell and CD4^+^ and CD8^+^ T-cell infiltration in a mouse tumor model [72].

Productive cGAS–STING signaling also requires adequate STING expression and activation-dependent trafficking from the endoplasmic reticulum to the Golgi apparatus. In CRC, radiotherapy can increase HMGCR expression and cholesterol biosynthesis in tumor cells. The resulting cholesterol accumulation retains STING in the endoplasmic reticulum and limits downstream type I interferon signaling, thereby reducing the immune-stimulatory effect of radiotherapy [73]. In CT26 and HCT116 cells, HMGCR inhibition restored radiotherapy-induced cGAS–STING signaling; in a CT26 syngeneic mouse model, lovastatin enhanced radiotherapy-associated CD8^+^ T-cell infiltration and tumor control in a cGAS–STING-dependent manner [73].

KRAS mutation can further suppress the capacity of CRC cells to signal through STING after cytosolic DNA sensing. In KRAS-mutant CRC, reduced miR-29b-3p expression increases DNMT3B abundance and promotes methylation of the TMEM173/STING promoter, leading to reduced STING expression and impaired radiotherapy-induced type I interferon production [74]. This mechanism was supported by analyses of KRAS-mutant CRC samples obtained before and after neoadjuvant chemoradiotherapy and by functional experiments in CRC cell and mouse models [74]. Restoration of miR-29b-3p increased STING signaling and improved radiotherapy-associated antitumor immune responses in KRAS-mutant tumors [74].

**Figure 2:**
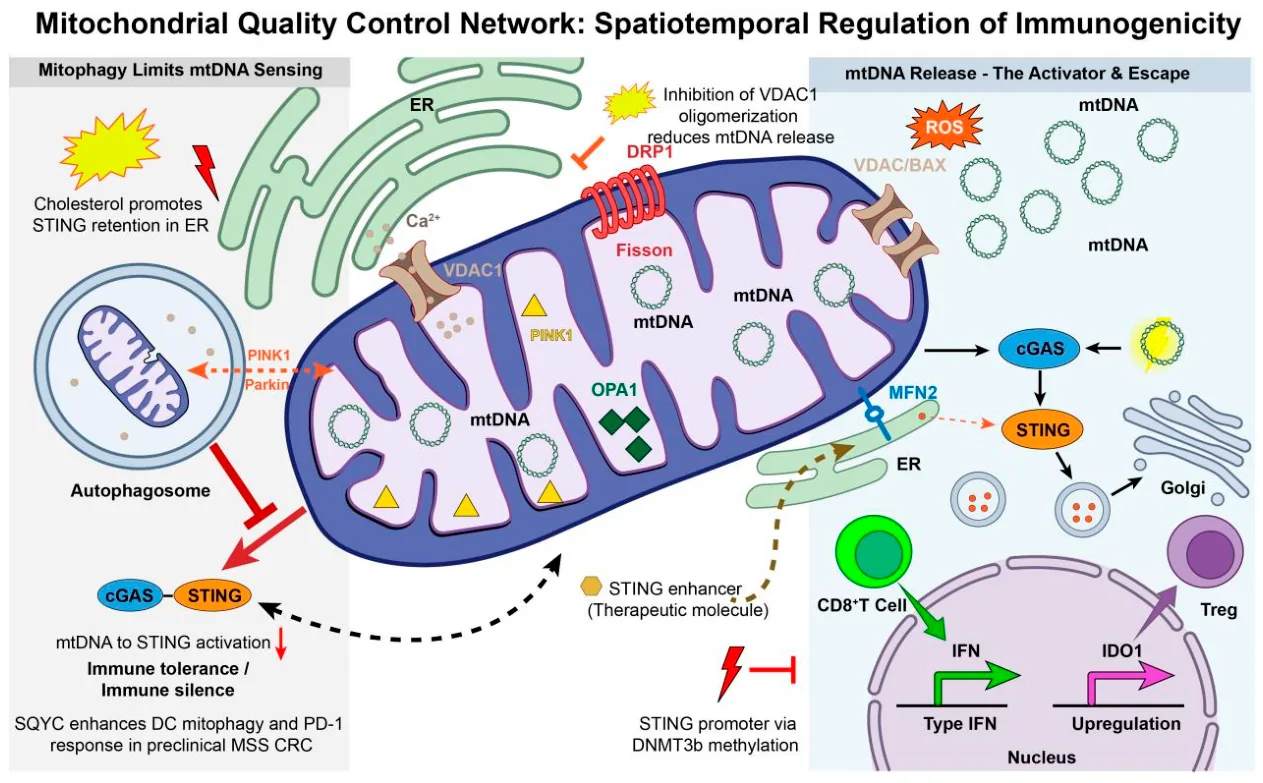
Mitochondrial quality control and mtDNA-dependent immune signaling in CRC. Mitophagy, mitochondrial dynamics, and ER–mitochondria contacts regulate mtDNA release and cGAS–STING signaling. Solid arrows indicate activation or transport; T-bars, inhibition; crosses, blockade; and dashed arrows, indirect effects.

Taken together, mtDNA-dependent immune signaling in CRC is governed by three sequential checkpoints: mtDNA stability, mitochondrial membrane escape, and the capacity of the cGAS–STING pathway to generate an interferon response. The ACADS–mito-DNMT1 axis regulates mtDNA methylation and release [70]; ferroptotic or VDAC-associated mitochondrial injury can promote cytosolic mtDNA accumulation [71,72]; and cholesterol- or KRAS-dependent mechanisms can subsequently restrict STING activation [73,74]. These regulatory layers help explain why mitochondrial stress does not uniformly produce an immune response and identify distinct points at which tumor-cell-intrinsic innate signaling can be restored. The mitochondrial quality-control and mtDNA-sensing pathways discussed in Section 4 are summarized in Figure 2.

## 5. Targeting Mitochondrial Metabolism for Immune Sensitization: Strategies and Prospects

The mechanisms discussed above identify several therapeutic opportunities to modify metabolic and mitochondrial determinants of immune suppression in CRC. Current approaches can be grouped into strategies that restrict tumor-cell nutrient utilization, alleviate metabolite-mediated immune suppression, restore the metabolic fitness of immune cells, enhance mtDNA-dependent innate immune signaling, or improve the delivery of metabolic interventions. Because these interventions differ in their primary cellular target, delivery method, combination partner, and level of supporting evidence, this section compares therapeutic strategies rather than reiterating the mechanisms described in the preceding sections.

**Figure 3:**
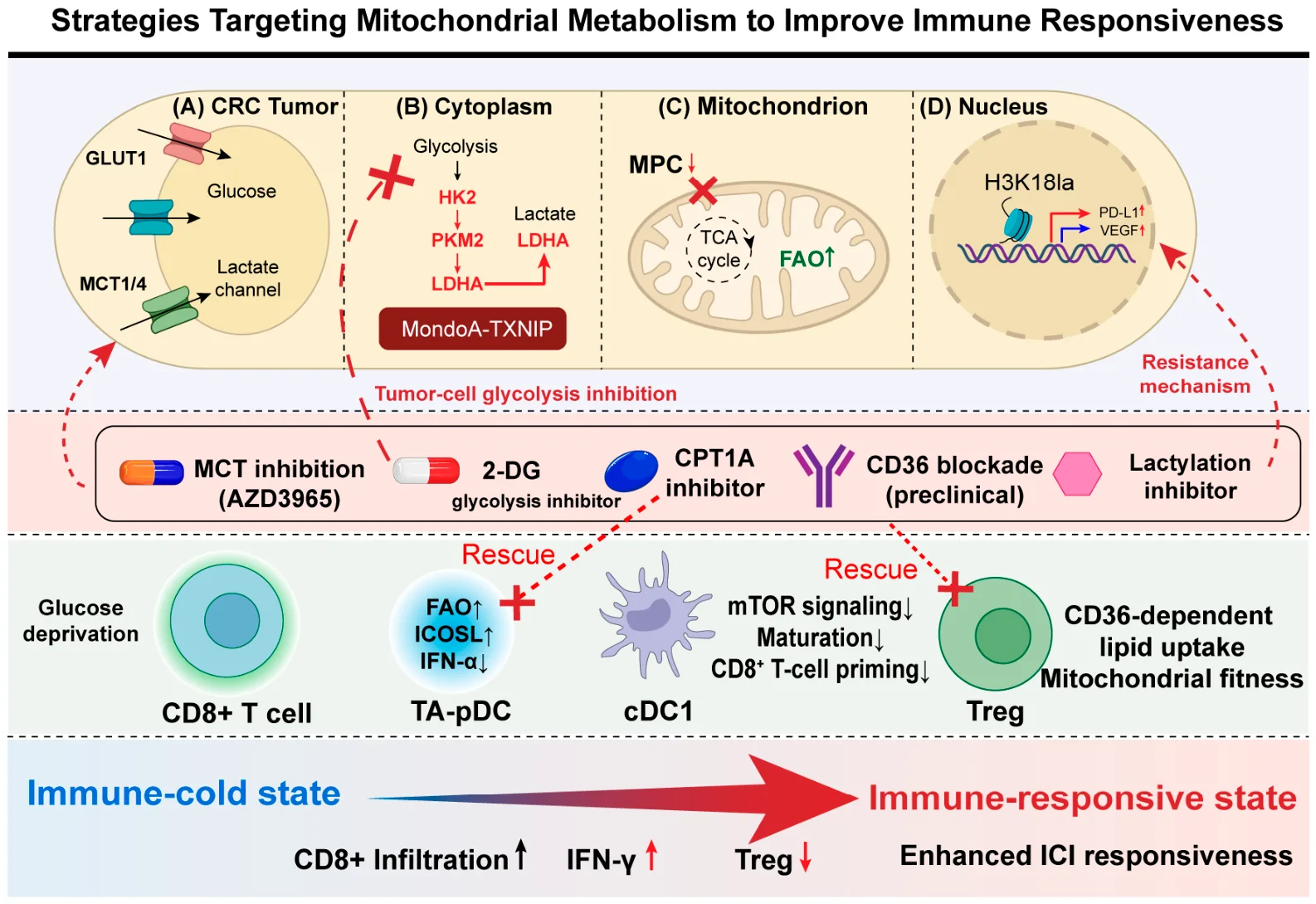
Strategies targeting mitochondrial metabolism to improve immune responsiveness in CRC. Glycolysis inhibition, MCT blockade, CPT1A inhibition, CD36 blockade, and targeting of lactylation may alleviate metabolic immunosuppression and enhance antitumor immune responses. Arrows indicate pathways or therapeutic effects; T-bars, inhibition; crosses, blockade; dashed lines, indirect effects; ↑/↓, increases/decreases.

In this review, delivery-system optimization refers to the use of nanoparticles, prodrugs, or other delivery platforms to improve exposure of a metabolic or immune modulator in the intended tumor or immune-cell compartment, enhance intracellular delivery or controlled release, and reduce off-target toxicity. Representative therapeutic strategies are summarized in Figure 3.

### 5.1. Targeting Glycolytic Dependence and Glucose Competition

Inhibition of glycolysis may reduce the high glucose demand of CRC cells and thereby alleviate metabolic constraints on infiltrating effector T cells [27,75]. In GSNOR-deficient CRC models, 2-deoxy-D-glucose (2-DG) reduced glycolytic activity, increased intratumoral CD8^+^ T-cell infiltration, and restored sensitivity to PD-1 blockade in vitro and in vivo [75]. These findings identify glycolytic dependence as a therapeutic vulnerability in GSNOR-deficient CRC. However, the antitumor effect of 2-DG is likely to depend on tumor genotype, metabolic context, and treatment schedule, because activated T cells also require glycolytic flux to support proliferation and effector function [27,40].

Nanoparticle-based delivery can also couple glycolytic suppression with immunogenic chemotherapy in CRC. Zhao et al. developed self-delivering DdLD nano-PROTACs assembled from the BRD4 degrader dBET57 and doxorubicin. In CT26 CRC cells, BRD4 degradation reduced c-Myc and PD-L1 expression, suppressed basal and compensatory glycolysis, and limited extracellular acidification, whereas doxorubicin promoted immunogenic cell death. In CT26 tumor-bearing mice, the formulation enhanced antitumor immune responses and inhibited tumor growth. This platform illustrates how delivery-system design can coordinate tumor-cell glycolytic suppression with immune activation, although its therapeutic effects have so far been demonstrated only in preclinical CRC models [76].

### 5.2. Targeting Metabolite-Driven Immune Suppression

Targeting amino acid and lactate metabolism may help alleviate metabolite-mediated immune suppression in CRC. The bifunctional prodrug T26 links a glutamine antagonist to a STING agonist through a tumor-microenvironment-responsive linker. In murine CRC models, T26 improved dendritic-cell maturation, increased CD8^+^ T-cell activation and intratumoral infiltration, and enhanced antitumor activity when combined with chemotherapy, immune checkpoint blockade, or antiangiogenic therapy [77]. Activity was also reported in patient-derived CRC organoids. This strategy is intended to combine suppression of tumor glutamine utilization with activation of antigen-presenting cells; its therapeutic activity remains preclinical.

Lactate-directed interventions can act at several points along the lactate pathway, including lactate production, MCT-dependent transport, extracellular acidification, HCAR1 signaling, and lactylation-associated transcriptional regulation. These targets should be compared according to the specific molecular node and experimental evidence rather than presented as a required sequential regimen. AZD3965 is a selective MCT1 inhibitor that was evaluated alongside selective MCT4 blockade in CRC spheroid–leukocyte co-cultures and MC38 mouse models. In these MCT4-dominant models, AZD3965 provided no additional benefit, whereas MCT4 inhibition improved immune-cell infiltration and enhanced the activity of PD-L1 blockade [34]. Whether combined inhibition of lactate production, transport, and lactylation provides additional benefit in CRC remains to be established in direct comparative studies.

Lipid-metabolic interventions may also weaken suppressive immune compartments. In colon-cancer specimens and mouse models, PPARγ-driven CPT1A expression increased fatty acid oxidation in tumor-associated plasmacytoid dendritic cells. CPT1A inhibition reduced ICOSL expression, limited pDC-associated Treg generation, improved CD8^+^ T-cell activity, and enhanced the antitumor effect of combined ICOSL and PD-L1 blockade [52]. CD36 blockade represents a related but distinct strategy. In preclinical tumor models, inhibition of CD36-mediated lipid uptake reduced the metabolic fitness and suppressive function of intratumoral Tregs [50]. Because CD36 is expressed in multiple tumor, immune, and stromal compartments, the cellular selectivity and therapeutic effects of CD36 blockade in CRC require further investigation.

### 5.3. Modulating Mitochondrial Quality Control and mtDNA Signaling

Mitochondrial quality-control interventions can enhance immune responsiveness through different cellular targets. In KRAS-mutant CRC cells, inhibition of DRP1 has been used to preserve mtDNA-dependent innate immune signaling during radiotherapy, with additional activity reported when combined with MEK inhibition in preclinical models [59]. By contrast, SQYC was reported to activate PINK1–Parkin-dependent mitophagy in dendritic cells, improve mitochondrial fitness and antigen-presentation-associated responses, and enhance sensitivity to PD-1 blockade in MSS CRC models [78]. Thus, DRP1 inhibition and PINK1–Parkin activation represent distinct therapeutic approaches: the former acts primarily on tumor-cell mitochondrial dynamics and radiotherapy-induced immunogenic signaling, whereas the latter is intended to support mitochondrial quality control and antigen presentation in dendritic cells. Both strategies remain supported by preclinical evidence, and their suitability depends on the cellular compartment and treatment combination being targeted.

In MSS/pMMR CRC, conversion from an immune-cold to an immune-responsive tumor state can be approached at several complementary levels. Increasing mitochondrial damage-associated signaling or restoring mtDNA–cGAS–STING activity may enhance type I interferon production and tumor-antigen sensing [56,70]. Improving mitochondrial fitness and antigen presentation in dendritic cells can strengthen the initiation of CD8^+^ T-cell responses [77,78], whereas inhibition of lactate transport, glutamine utilization, or suppressive lipid metabolism may reduce metabolic constraints on immune-cell infiltration and effector function [34,77,79]. Delivery systems that concentrate these interventions within tumor cells or defined immune-cell populations may further improve their selectivity. Evidence of immune sensitization should therefore include coordinated changes in innate immune activation, antigen presentation, effector-cell recruitment and function, and response to checkpoint blockade, rather than increased T-cell numbers alone.

### 5.4. Delivery Systems and Gene-Editing Approaches

Delivery platforms can improve the local activity of mitochondrial or organelle-targeted interventions by increasing tumor accumulation and coordinating the delivery of agents with complementary mechanisms. The hyaluronic acid-coated HA/pCe6-Ato nanoplatform was designed to target CD44-expressing CRC stem-like cells and co-deliver the endoplasmic-reticulum-targeting photosensitizer pCe6 and atovaquone, an inhibitor of mitochondrial respiratory-chain complex III. In CRC cell and mouse-tumor models, photoactivation induced ER stress, mitochondrial dysfunction, reactive oxygen species production, and tumor-cell death. These changes were accompanied by reduced stemness-associated features, lower expression of immunosuppressive mediators, and increased infiltration of cytotoxic immune cells [80]. This study illustrates how dual-organelle targeting may improve local treatment responses; its immune-sensitizing activity remains supported by preclinical evidence.

Gene-editing delivery represents a separate strategy for targeting mitochondrial determinants of chemoresistance. An orally administered CRISPR–Cas9 nanocomplex was developed to disrupt TRAP1, which encodes a mitochondrial chaperone, in CRC cells. TRAP1 disruption promoted cyclophilin-D translocation and sustained opening of the mitochondrial permeability transition pore, thereby enhancing chemotherapy-induced necrotic cell death and activation of the tumor immune microenvironment. In orthotopic, chemoresistant, and spontaneous CRC mouse models, oral delivery of the TRAP1-targeting CRISPR–Cas9 system enhanced the antitumor activity of chemotherapy [81]. Although this work provides a proof of concept for oral gene-editing delivery in CRC, editing specificity, long-term safety, host immune responses to Cas9, and scalability require assessment before clinical translation. The principal therapeutic strategies discussed in this section are compared in Table 1 according to their molecular targets, primary cellular compartments, supporting experimental models, and major translational limitations.

### 5.5. Translational Challenges and Future Directions

Despite the therapeutic rationale for targeting mitochondrial metabolism, clinical translation remains limited by the narrow therapeutic window of interventions that impair mitochondrial respiration. Because oxidative phosphorylation is required in normal tissues with high energy demand, including the nervous system, skeletal muscle, heart, and hematopoietic cells, systemic inhibition may cause on-target toxicity before sufficient antitumor exposure is achieved. IACS-010759, a potent inhibitor of mitochondrial electron-transport-chain complex I, illustrates this challenge. Although it showed substantial antitumor activity in oxidative-phosphorylation-dependent preclinical models, two phase I trials in relapsed/refractory acute myeloid leukemia and advanced solid tumors identified elevated blood lactate and neurotoxicity as dose-limiting toxicities. Target exposure could not be maintained, no recommended phase II dose was established, and both trials were discontinued [82]. These studies included patients with advanced CRC but did not establish a CRC-specific clinical benefit. The respiratory-chain site and mode of inhibition are relevant to both therapeutic activity and toxicity. IACS-010759 directly inhibits NADH oxidation by complex I and was administered systemically, whereas atovaquone inhibits electron transfer through complex III and, in the HA/pCe6-Ato system described above, was incorporated into a tumor-targeted delivery platform. These examples illustrate two different approaches to respiratory-chain modulation: systemic inhibition of a defined electron-transport-chain complex and localized delivery intended to restrict respiratory inhibition to the tumor. Direct comparisons of their selectivity and therapeutic index in CRC have not yet been established [80,82].

Predictive biomarkers are needed to identify patients whose tumors are most likely to depend on a specific mitochondrial program. Candidate biomarkers include mitochondrial functional states, such as relative dependence on oxidative phosphorylation or glycolysis; genomic alterations that affect mitochondrial stress signaling; and metabolic or immune features identified by transcriptomic, proteomic, or spatial analyses. mtDNA mutations, copy-number changes, and heteroplasmy patterns may also be informative [67,68,69], but their association with response to mitochondrial-targeted therapy in CRC has not yet been prospectively validated. Biomarker development should therefore integrate tumor genotype, mitochondrial function, metabolic phenotype, and immune context rather than rely on a single mitochondrial marker. Current precision treatment in CRC provides a practical framework for incorporating molecular and pharmacogenetic testing into therapeutic decision-making. Tumor testing for RAS and BRAF alterations and for MSI or mismatch-repair status is used to guide the selection of anti-EGFR therapy, BRAF-targeted combinations, and immune checkpoint inhibitors, respectively [83]. Pharmacogenetic testing can additionally inform treatment safety: reduced-function DPYD variants are associated with severe fluoropyrimidine toxicity [84], whereas UGT1A1 variants can increase the risk of irinotecan-associated neutropenia and may support dose adjustment in selected patients [85]. Although these biomarkers do not directly predict sensitivity to mitochondria-targeted agents, they illustrate how pretreatment testing can connect molecular characteristics with drug selection and dosing. A comparable strategy for mitochondrial therapies will require prospectively validated biomarkers that identify both tumor dependence on the targeted metabolic pathway and the risk of toxicity in normal tissues.

Delivery-system optimization remains a major challenge, particularly for agents intended to act within mitochondria [86]. Effective mitochondrial delivery requires sufficient accumulation in the relevant tumor or immune-cell compartment, cellular uptake, escape from endosomal sequestration, access to the mitochondrial surface or matrix, and retention of pharmacological activity at the intended sub-mitochondrial site. For matrix-directed agents, membrane-potential-dependent accumulation may be useful, whereas interventions targeting mitochondrial outer-membrane proteins, permeability transition, or mtDNA release may require different delivery properties. These requirements must be balanced against off-target accumulation in normal tissues and the potential disruption of mitochondrial function in immune cells [86].

**Table 1:** Representative therapeutic strategies targeting mitochondrial metabolism to improve immune responsiveness in colorectal cancer.

Strategy/Representative Agent	Metabolic Target or Rationale	Clinical Context or Experimental Model	Selected Outcome or Status	Translational Interpretation
Metformin + nivolumab	Mitochondrial complex I-associated metabolic modulation combined with PD-1 blockade	Phase II; treatment-refractory MSS metastatic CRC; NCT03800602 [87]	No objective responses among 18 evaluable patients; two achieved stable disease; trial stopped for futility	CRC-specific clinical evidence, but no demonstrated efficacy; illustrates that nonspecific metabolic modulation alone may be insufficient
ME-344 + bevacizumab	OXPHOS inhibition after anti-VEGF-induced metabolic adaptation	Phase 1b; refractory metastatic CRC; 23 patients; NCT05824559 [88]	No objective responses; 39% achieved stable disease; median PFS 1.9 months	CRC-specific clinical evidence with limited disease control; exposure and patient selection require optimization
Telaglenastat (CB-839) + capecitabine	Glutaminase inhibition to restrict glutamine-dependent anaplerosis	Phase I; fluoropyrimidine-resistant, PIK3CA-mutant CRC and other solid tumors; NCT02861300 [89]	No objective responses; stable disease in 10/16 patients [89].	Demonstrates clinical feasibility of glutamine-metabolism targeting, but CRC benefit is unconfirmed
IACS-010759	Systemic inhibition of mitochondrial respiratory-chain complex I	Phase I; advanced solid tumors and relapsed/refractory AML; not CRC-specific [82]	Elevated blood lactate and neurotoxicity limited sustained target exposure; no recommended phase II dose established	Clinical evidence highlights the narrow therapeutic window of systemic OXPHOS inhibition; no established CRC benefit
T26	Tumor-directed glutamine antagonism combined with STING activation in antigen-presenting cells	Murine CRC models and patient-derived CRC organoids [77]	Enhanced DC maturation and CD8^+^ T-cell responses; evaluated with chemotherapy and immunotherapy	Representative dual-function preclinical strategy; pharmacokinetics, cellular selectivity, and systemic safety remain unresolved
CD36 blockade (PLT012)	CD36-dependent lipid uptake and mitochondrial fitness in intratumoral Tregs	MC38-based CRC liver-metastasis models [79]	Reduced Treg metabolic fitness and improved the effect of PD-1 blockade in preclinical models	CRC evidence remains preclinical; activity in patients with CRC has not been established
2-DG + anti-PD-1	Tumor-cell glycolysis inhibition and reduction of glucose competition	GSNOR-deficient CRC cell and mouse models [75]	Increased intratumoral CD8^+^ T-cell infiltration and improved tumor control	Preclinical; systemic glycolysis inhibition may also impair activated immune cells and requires biomarker-based selection
Tumor-targeted nanoplatforms	Localized delivery targeting tumor glycolysis or coordinated ER–mitochondrial stress	CT26 CRC cells and mouse models; CRC stem-like cells and mouse-tumor models [76,80]	Suppressed tumor glycolysis or cancer stemness and enhanced antitumor immune responses in preclinical CRC models	May improve spatial and cellular selectivity, but biodistribution, manufacturing reproducibility, treatment activation, and long-term safety remain barriers

Abbreviations: 2-DG, 2-deoxy-D-glucose; AML, acute myeloid leukemia; CD8, cluster of differentiation 8; CRC, colorectal cancer; DC, dendritic cell; ER, endoplasmic reticulum; GSNOR, S-nitrosoglutathione reductase; MSS, microsatellite stable; OXPHOS, oxidative phosphorylation; PD-1, programmed cell death protein 1; PFS, progression-free survival; STING, stimulator of interferon genes; Treg, regulatory T cell; VEGF, vascular endothelial growth factor.

Future studies should integrate metabolic profiling, spatial analysis of tumor–immune interactions, and pharmacodynamic monitoring to match each intervention with the relevant molecular subtype and immune context. Rather than broadly suppressing mitochondrial function, the most feasible strategies may selectively target defined metabolic dependencies and combine them with radiotherapy, chemotherapy, immune checkpoint blockade, or innate immune agonists at doses that preserve systemic mitochondrial function. Table 1 compares representative mitochondrial metabolism-targeted strategies according to their molecular targets, primary cellular compartments, supporting experimental models, and major translational limitations.

## 6. Conclusions

Mitochondrial metabolic reprogramming contributes to immune evasion in CRC through interconnected changes in nutrient utilization, mitochondrial quality control, and mitochondria-associated immune signaling. Altered glucose, glutamine, lactate, and lipid metabolism can reshape the metabolic conditions experienced by tumor-infiltrating T cells, dendritic cells, macrophages, and regulatory T cells. In parallel, mitochondrial dynamics, mitophagy, mitochondrial membrane integrity, and mtDNA release regulate the availability of mitochondrial danger signals and the activity of innate immune pathways, particularly cGAS–STING signaling.

The consequences of these processes depend on the cellular compartment and experimental context. A metabolic alteration that supports tumor-cell survival may impair immune-cell function, whereas controlled mitochondrial stress or mtDNA release may enhance antitumor immune recognition. Therefore, therapeutic strategies should be designed around defined molecular dependencies and target-cell populations rather than broad suppression of mitochondrial function.

Most mitochondria-directed immune-sensitization strategies in CRC remain supported by cell-based, organoid, and animal studies. Future work should validate candidate biomarkers, define pharmacodynamic markers of target engagement, and determine whether tumor-selective delivery or rational combination regimens can improve the therapeutic index. Integrating metabolic profiling with spatial and immune analyses may help identify CRC subgroups in which mitochondrial interventions can enhance responsiveness to chemotherapy, radiotherapy, or immune checkpoint blockade.

## Data Availability

No new data were created or analyzed in this study. Data sharing is not applicable to this article.
